# Human neuronal changes in brain edema and increased intracranial pressure

**DOI:** 10.1186/s40478-016-0356-x

**Published:** 2016-08-04

**Authors:** Nóra Faragó, Ágnes Katalin Kocsis, Csilla Braskó, Sándor Lovas, Márton Rózsa, Judith Baka, Balázs Kovács, Katalin Mikite, Viktor Szemenyei, Gábor Molnár, Attila Ozsvár, Gáspár Oláh, Ildikó Piszár, Ágnes Zvara, Attila Patócs, Pál Barzó, László G. Puskás, Gábor Tamás

**Affiliations:** 1 MTA-SZTE Research Group for Cortical Microcircuits of the Hungarian Academy of Sciences, Department of Physiology, Anatomy and Neuroscience, University of Szeged, Közép fasor 52, Szeged, H-6726 Hungary; 2 Laboratory of Functional Genomics, Department of Genetics, Biological Research Center, Hungarian Academy of Sciences, Temesvári krt. 62, Szeged, H-6726 Hungary; 3 Avidin Ltd., Alsó kikötő sor 11, Szeged, H-6726 Hungary; 4 MTA Lendület Hereditary Endocrine Tumors Research Group, Semmelweis University, Szentkirályi u. 46, Budapest, H-1088 Hungary; 5 Department of Neurosurgery, University of Szeged, Semmelweis u. 6, Szeged, H-6725 Hungary

## Abstract

Functional and molecular changes associated with pathophysiological conditions are relatively easily detected based on tissue samples collected from patients. Population specific cellular responses to disease might remain undiscovered in samples taken from organs formed by a multitude of cell types. This is particularly apparent in the human cerebral cortex composed of a yet undefined number of neuron types with a potentially different involvement in disease processes. We combined cellular electrophysiology, anatomy and single cell digital PCR in human neurons identified in situ for the first time to assess mRNA expression and corresponding functional changes in response to edema and increased intracranial pressure. In single pyramidal cells, mRNA copy numbers of *AQP1*, *AQP3*, *HMOX1*, *KCNN4*, *SCN3B* and *SOD2* increased, while *CACNA1B*, *CRH* decreased in edema. In addition, single pyramidal cells increased the copy number of *AQP1*, *HTR5A* and *KCNS1* mRNAs in response to increased intracranial pressure. In contrast to pyramidal cells, *AQP1*, *HMOX1*and *KCNN4* remained unchanged in single cell digital PCR performed on fast spiking cells in edema. Corroborating single cell digital PCR results, pharmacological and immunohistochemical results also suggested the presence of *KCNN4* encoding the α-subunit of KCa3.1 channels in edema on pyramidal cells, but not on interneurons. We measured the frequency of spontaneous EPSPs on pyramidal cells in both pathophysiological conditions and on fast spiking interneurons in edema and found a significant decrease in each case, which was accompanied by an increase in input resistances on both cell types and by a drop in dendritic spine density on pyramidal cells consistent with a loss of excitatory synapses. Our results identify anatomical and/or physiological changes in human pyramidal and fast spiking cells in edema and increased intracranial pressure revealing cell type specific quantitative changes in gene expression. Some of the edema/increased intracranial pressure modulated and single human pyramidal cell verified gene products identified here might be considered as novel pharmacological targets in cell type specific neuroprotection.

## Introduction

Brain edema and an increase in intracranial pressure (ICP) might result from a number of insults including traumatic brain injury, cerebral ischemia, hypoxia, infection, brain tumors, and neuroinflammation [35]. The localization and extent of brain damage correlates with the neurological outcome, but permanent deterioration and death are also causally linked to edema or infarct of the perilesional tissue. The mainstream treatment for edema and increased ICP has been unchanged during the last 35–40 years (cerebrospinal fluid drainage, sedation, hyperventilation, osmotherapy, steroids, barbiturate and decompressive craniectomy as an ultimum refugium) [36, 44] and no target designed pharmacological treatment specifically lowering brain edema is available to patients [36, 44].

Advances in unbiased large scale molecular approaches are proven to be successful in pinpointing individual or multiple genes associated with a disease [1]. Omics based assessment of human pathological conditions is usually based on tissue samples containing a mixture of cell types. Blood samples are relatively easily sorted into different cell classes prior to molecular characterization, but similarly automatized methods are not readily available for biopsies taken from solid tissues [8, 28]. The cerebral cortex, composed of several distinct classes of neurons, glial cells and cell types forming the vasculature, is particularly challenging from this respect [22, 46] and pioneering laser-capture microdissection studies confirm cell type specific expression patterns in disease [33]. Previous work showed that neuron classes recorded in acute brain slices made from biopsies of the cerebral cortex can be classified based on their electrophysiological features [21, 24, 34, 43]. Such recordings allow intracellular labeling and anatomical analysis of the recorded cells further refining the identification of cell types. Moreover, whole cell patch clamp recordings provide an opportunity for harvesting cytoplasm from electrophysiologically and morphologically identified neurons [25, 41]. Quantification of mRNA with single molecule precision from the cytoplasm collected from individual neurons [13, 23] could provide cell type specific information in addition to tissue level changes, and, in turn, electrophysiological recordings can further validate the function of therapeutically relevant genes in identified neurons [23]. We present a workflow for detecting disease related mRNA expression changes in biopsies of the cerebral cortex which is capable of validating the alterations predicted at the tissue level with the precision of individual mRNA molecules in single neurons combined with functional validation in identified cells targeted in situ.

## Materials and methods

### Patients

We analysed neocortical tissue surgically removed from patients (*n* = 37, *n* = 22 female and *n* = 15 male, aged 49 ± 14 years) in a course of two years as part of the treatment protocol for aneurysms, brain tumors and/or increased intracranial pressure. Tissue blocks were removed from prefrontal (*n* = 22), temporal (*n* = 8) and parietal (*n* = 7) areas. Patients were grouped according to the presence of edema based on MRI scans showing hypointense signal in T1 weighted sequences and hyperintense signal in T2 weighted sequences [35] and according to measurements of ICP using Codman Microsensor™ ventricular and/or parenchymal kit [5]. Patient group 1 (Control, *n* = 11, *n* = 5 female and *n* = 6 male, aged 46 ± 15 years) had no detectable edema or increase in ICP. These patients were diagnosed with unruptured aneurisms (*n* = 2) and benign (*n* = 56) or malignant (*n* = 3) tumors (WHO grade II) detected serendipitously at early stages. Patient group 2 (Edema, *n* = 18, *n* = 12 female and *n* = 6 male, aged 52 ± 11 years) showed edema on MRI scans and 0–14 Hgmm ICP. These patients were diagnosed with primary benign (*n* = 3) or malign brain tumors (*n* = 12), tumor metastases (*n* = 2) or abscess (*n* = 1). Patient group 3 (Pressure, *n* = 10, *n* = 7 female and *n* = 3 male, aged 42 ± 16 years) showed no edema on MRI scans and had 21–43 Hgmm ICP. These patients suffered from hydrocephalus caused by benign (*n* = 4) or malign (*n* = 4) brain tumors, by a colloid cyst (*n* = 1) or by Arnold-Chiari syndrome (*n* = 1).

### Electrophysiology

All procedures were performed according to the Declaration of Helsinki with the approval of the University of Szeged Ethical Committee. Human slices were derived from material which had to be removed according to treatment protocols from prefrontal regions (gyrus frontalis superior and medialis of either hemispheres and right gyrus frontalis inferior, the left gyrus frontalis inferior was avoided in order to keep Broca’s area intact), from temporal regions of both hemispheres (gyrus temporalis superior, medialis and inferior) and from parietal regions with written informed consent patients prior to surgery over the last three years. Patients having a record of epileptic seizures and with drugs related to epilepsy were excluded from the study. Anesthesia was induced with intravenous midazolam and fentanyl (0.03 mg/kg, 1–2 μg/kg respectively). A bolus dose of propofol (1–2 mg/kg) was administered intravenously. To facilitate endotracheal intubation, the patient received 0.5 mg/kg rocuronium. After 120 s the trachea was intubated and the patient was ventilated with a mixture of O_2_ and N_2_O at a ratio of 1:2. Anesthesia was maintained with sevoflurane at minimum alveolar concentration volume of 1.2–1.5. Blocks of tissue were immersed into ice cold solution containing (in mM) 130 NaCl, 3.5 KCl, 1 NaH_2_PO_4_, 24 NaHCO_3_, 1 CaCl2, 3 MgSO_4_, 10 d (+)-glucose, saturated with 95 % O_2_ and 5 % CO_2_ in the operating theatre, sliced at a thickness of 350 μm with a vibrating blade microtome (Microm HM 650 V) and were incubated at room temperature for 1 h in the same solution. The solution used during recordings differed only in that it contained 2 mM CaCl_2_ and 1.5 mM MgSO_4_. Recordings were obtained at ~36 °C from up to four concomitantly recorded cells visualized in layer 2/3 by infrared differential interference contrast videomicroscopy at depths 60–130 μm from the surface of the slice. Micropipettes (5–7 MΩ) were filled with an intracellular solution containing 126 mM K-gluconate, 4 mM KCl, 10 mM HEPES, 10 mM creatine phosphate, 8 mM biocytin; pH 7.25; 300 mOsm supplemented with RNase Inhibitor (1U/μl, Life Technologies) to prevent any RNA degradation. Access resistance was monitored with −10 mV voltage steps in between experimental epochs and data collection was terminated if access resistance exceeded 30 MΩ. Signals were filtered at 8 kHz, digitized at 16 kHz and analyzed with PULSE software. Membrane properties of human neurons or polysynaptic events did not show significant changes for up to 20 h after slicing, but recordings included in the analysis were arbitrarily terminated 15 h after slice preparation. Traces shown are single sweeps (firing patterns) or averages or of 50–100 consecutive episodes (pharmacology). The effects during drug application were tested following 5 min wash-in periods. Visualization of biocytin and microscopy was performed as described earlier [24].

### Single cell reverse transcription and digital PCR

At the end of electrophysiological recordings, the intracellular content was aspirated into the recording pipettes by application of a gentle negative pressure while maintaining the tight seal. Pipettes were then delicately removed to allow outside-out patch formation, and the content of the pipettes (~1.5 μl) was expelled into a low-adsorbtion test tube (Axygen) containing 0.5 μl SingleCellProtectTM (Avidin Ltd. Szeged, Hungary) solution in order to prevent nucleic acid degradation and to be compatible with direct reverse transcription reaction. Samples were snap-frozen in liquid nitrogen and stored or immediately used for reverse transcription. The reverse transcription of the harvested cytoplasm was carried out in two steps. The first step was done for 5 min at 65 °C in a total reaction volume of 5 μl containing 2 μl intracellular solution and SingleCellProtectTM mix with the cytoplasmic contents of the neuron, 0.3 μl TaqMan Assays, 0.3 μl 10 mM dNTPs, 1 μl 5× first-strand buffer, 0.3 μl 0.1 mol/L DTT, 0.3 μl RNase inhibitor (Life Technologies) and 100 U of reverse transcriptase (Superscript III, Invitrogen). The second step of the reaction was carried out at 55 °C for 1 h and then the reaction was stopped by heating at 75 °C for 15 min. The reverse transcription reaction mix was stored at −20 °C until PCR amplification.

For digital PCR analysis, half of the reverse transcription reaction mixture (2.5 μl), 2 μl TaqMan Assays (Life Technologies), 10 μl OpenArray Digital PCR Master Mix (Life Technologies) and nuclease free water (5.5 μl) were mixed in a total volume of 20 μl. The mixture was evenly distributed on an OpenArray plate. RT mixes were loaded into 4 wells of a 384-well plate from which the OpenArray autoloader transferred the cDNA master mix by capillary action into 256 nanocapillary holes (4 subarrays) on an OpenArray plate. Processing of the OpenArray slide, cycling in the OpenArray NT cycler and data analysis were done as previously described [13]. For our dPCR protocol amplification, reactions with *CT* confidence values below 100 as well as reactions having *CT* values less than 23 or greater than 33 were considered primer dimers or background signals, respectively, and excluded from the data set.

### RNA preparation, amplification and labeling

Total RNA was purified from each sample using an RNA purification kit (Macherey Nagel, Düren, Germany) according to the manufacturer’s instructions. At a final concentration of 0.8 U/μl, an RNase inhibitor (Fermentas, Lithuania) was added to the samples. RNA quantity was determined using Agilent Bioanalyzer 2100 NanoDrop 3.1.0. RNA samples were stored at −80 °C before used. An aliquot of the total RNA (1 μg) was amplified with the AminoAllyl MessageAmpTM II aRNA Amplification Kit (Ambion, USA) according to the manufacturer’s instructions. Six microgram aminoallyl-modified amplified RNA (aaRNA) was labeled with Cy3 dye according to the manufacturer’s instructions (Ambion, USA) then purified. The dye incorporation rate and the labeled aRNA concentration were detected using NanoDrop 3.1.0. The incorporation rate of the samples was 30–60 dye molecules per 1000 nucleotides.

### Microarray hybridization, raw data extraction and analysis

The human oligonucleotide microarray (Whole Human Genome Microarray, 4 × 44 K, G4112F, Design ID 014850) from Agilent Technologies Inc. (Santa Clara, CA, USA) was used to determine gene expression changes. 825 ng of Cy3 labeled aaRNA, 11 μl 10× Blocking Agent and 2.2 μl 25× Fragmentation Buffer were mixed together in a final volume of 55 μl and incubated at 60 °C for 30 min then 55 μl 2× GEx Hybridization Buffer were added to each sample to stop the fragmentation reaction. All these steps were done using Gene Expression Hybridization Kit of Agilent Technologies according to the manufacturer’s instructions. 100 μl of these mixes were used to fill the 4-plex backing slides and the microarray was placed onto it. This “hybridization sandwich” was assembled in an Agilent microarray hybridization chambers. The chambers were then loaded into a hybridization rotator rack (~5 rpm) and incubated at 65 °C for 17 h. After hybridization the slides were washed in Wash buffer 1 from Agilent Technologies at room temperature for 1 min than in Wash buffer 2 at 37 °C for another 1 min before scanning. Each array was scanned at 543 nm (for Cy3 labeling) in Agilent Scanner (G2505B) using the extended dynamic range function with 5 μm resolution. Output image analysis and feature extraction was done using Feature Extraction 9.5.1 software using the single-color gene expression protocol (GE1_1100_Jul11).

All the raw data extracted by Feature Extraction software was statistically analyzed by GeneSpringGX 13.0 software from Agilent Technologies. Percentile Shift Normalization method was applied to globally normalize all the spot intensities. All the ratios were calculated from the average signal intensities in each groups (control *n* = 9, odema *n* = 12 and press *n* = 6) and one-way ANOVA with unequal variances (Welch) test with a twofold change cutoff was applied to determine which genes showed significantly altered expression levels. If more than a half of the data points in an experimental group had a “not detected” value - which corresponded to a not significant data point (not significantly above the background) - were excluded. Gene expression ratios with *p* value of < 0.05 and log2 ratio of < −1 or log2 ratio of >1 (2 fold change) were considered as repression or overexpression respectively in gene activity. The MIAME formatted microarray data are deposited to EMBL - European Bioinformatics Institute ArrayExpress system [http://www.ebi.ac.uk/microarray-as/ae/] with the Accession Number: E-MTAB-3678.

### Quantitative real-time PCR (QRT-PCR)

In order to validate gene expression changes obtained by DNA microarray, QRT-PCR was performed on an Exicycler-96 instrument (Bioneer) with gene-specific TaqMan assays and TaqMan protocol to monitor gene expression. 1 μg of total RNA was reverse transcribed using the High-Capacity cDNA Archive Kit (Life Technologies) according to the manufacturer’s instructions in a final volume of 30 μL. After dilution with 60 μL of water, 1 μL of the diluted reaction mix was used as template in the QRT-PCR with FastStart TaqMan Probe Master mix (Roche Applied Science) with the following protocol: 10 min at 95 °C followed by 55 cycles of 95 °C for 10 s and 60 °C for 30 s. The fluorescence intensity of FAM dye was detected after each amplification step. Relative expression ratios were calculated as normalized ratios to rat GAPDH housekeeping gene. The final relative gene expression ratios were calculated as delta-delta Ct values. Fold change refers to 2^-ΔΔCt^ (in the case of up-regulated genes) and – (1/2^-ΔΔCt^) (in the case of down-regulated genes).

### Immunohistochemistry

Cortical sections were prepared as described above for electrophysiology at 320–500 μm thickness and immersed immediately in a fixative containing 1 % paraformaldehyde and 0.2 % (w/v) picric acid dissolved in 0.1 M phosphate buffer (pH 7.3–7.4) for 4 h. Slices were resectioned at 50 μm thickness and a proteolytic antigen retrieval method was used to localise membrane–bound epitopes. Briefly, the tissue sections were incubated at 37 °C for 10 min in 0.1 M phosphate buffer followed by 5 min in 0.2 M HCl containing preheated 0.2 mg/ml pepsin (Dako), then sections were washed in 0.1 M phosphate buffer for 2 × 10 and 1 × 30 min. For the comparison of KCNN4 expression in human control and cerebral edema cortex sections were kept in the same vial to ensure equal conditions throughout the procedure. Following several washes in tris-buffered saline, non-specific binding was blocked by incubating sections in 20 % dried milk and 0.05 % Tween 20 for 2 h at room temperature. The tissue sections were incubated with Rabbit-KCNN4 1:300 (Alomone, APC-064) or Rabbit-KCNN4 1:100 (Thermo PA5-33875) primary antibody diluted in tris-buffered saline, 2 days at 4 °C. After several washes with TBS, sections were incubated in Alexa 488 -donkey anti-rabbit (Jackson) diluted 1∶500, for 2 h at room temperature. Sections were washed 3 times in tris-buffered saline and then in 0.1 M phosphate buffer and mounted in Vectashield mounting medium (Vector Laboratories, Burlingame, CA).

### Statistics

Data are given as mean ± S.D., datasets were statistically compared using one-way ANOVA or Kruskal-Wallis test and Wilcoxon test was used for pharmacological experiments with SPSS software (IBM), differences were accepted as significant if *p* ≤ 0.05.

## Results

In order to detect genes potentially regulated by edema and increased ICP relative to control conditions, we performed DNA microarray analysis in homogenized tissue samples of the gray matter derived from the three groups of patients (*n* = 9, 12 and 6 from Control, Edema and Pressure groups, respectively). This preliminary analysis indicated 525 and 148 upregulated and 1119 and 168 downregulated genes in the edemic and ICP groups, respectively (one way Welch ANOVA, *p* < 0.05, see uploaded data). Based on these suspected changes and potential clinical relevance, we selected 17 genes from the Control versus Edema groups and 10 genes from the Control versus Pressure group comparison for quantitative real-time PCR (QRT-PCR) validation (Table 1). QRT-PCR data were in agreement with microarray predictions in 12 (67 %) and 4 (40 %) cases in samples taken from the Edema and Pressure groups, respectively.

**Table 1:** Comparison of microarray and QPCR measurements on homogenates of cortical gray matter derived from the Control versus Edema and Control versus Pressure groups of patients

Gene symbol	Microarray				QPCR			
	Log2 change ([odema] vs [cont])	*p* value (OneWay Welch ANOVA)	Fold change	Regulation	Log2 change (ΔΔCt [odema] vs [cont])	*p* value (Student’s ttest)	Fold change	Regulation
	Control versus Edema							
*AQP1*	1.71	0.0383	3.26	up	5.20	0.0000	36.85	up
*AQP3*	2.28	0.0007	4.87	up	3.02	0.0005	8.11	up
*CACNA1B*	−1.06	0.0282	−2.09	down	−2.71	0.0025	−6.55	down
*CRH*	−2.82	0.0000	−7.06	down	−3.59	0.0046	−12.00	down
*DRD5*	−1.17	0.0037	−2.26	down	−3.32	0.0045	−9.98	down
*GABRA4*	−2.12	0.0091	−4.36	down	1.02	0.1008	2.03	up
*GNRH1*	−0.35	0.0629	−1.28	nc	−2.14	0.0309	−4.42	down
*GRIA3*	−1.34	0.0132	−2.53	down	−2.59	0.0058	−6.03	down
*GRID1*	−1.12	0.0230	−2.17	down	0.42	0.6465	1.34	nc
*HMOX1*	1.68	0.0013	3.21	up	2.57	0.0035	5.93	up
*KCNN4*	1.58	0.0002	2.99	up	1.63	0.0434	3.09	up
*KCTD12*	1.06	0.0025	2.09	up	0.34	0.6573	1.27	nc
*NEUROD6*	−2.37	0.0020	−5.17	down	−0.16	0.8805	0.89	nc
*NPY1R*	−1.61	0.0122	−3.06	down	−1.55	0.0069	−2.93	down
*SCN3B*	1.33	0.0235	2.52	up	2.07	0.0057	4.20	up
*SOD2*	1.01	0.0030	2.02	up	3.33	0.0014	10.06	up
	Control versus Pressure							
*AQP1*	0.13	0.0383	1.09	nc	4.78	0.0015	27.38	up
*GRIK4*	0.18	0.0237	1.13	nc	−2.44	0.0144	−5.41	down
*HTR5A*	1.20	0.0178	2.29	up	1.44	0.0221	2.71	up
*KCNAB3*	0.90	0.0008	1.86	up	1.52	0.0279	2.87	up
*KCNS1*	1.09	0.0200	2.14	up	3.05	0.0006	8.25	up
*KCNS3*	1.09	0.0024	2.13	up	0.77	0.5766	1.71	nc
*PVALB*	1.16	0.0011	2.24	up	2.02	0.0056	4.04	up
*SLC2A3*	−0.37	0.0231	−1.30	nc	−1.21	0.0267	−2.31	down
*SLC2A9*	−0.22	0.4318	−1.16	nc	−2.01	0.0056	−4.02	down

**Fig. 1:**
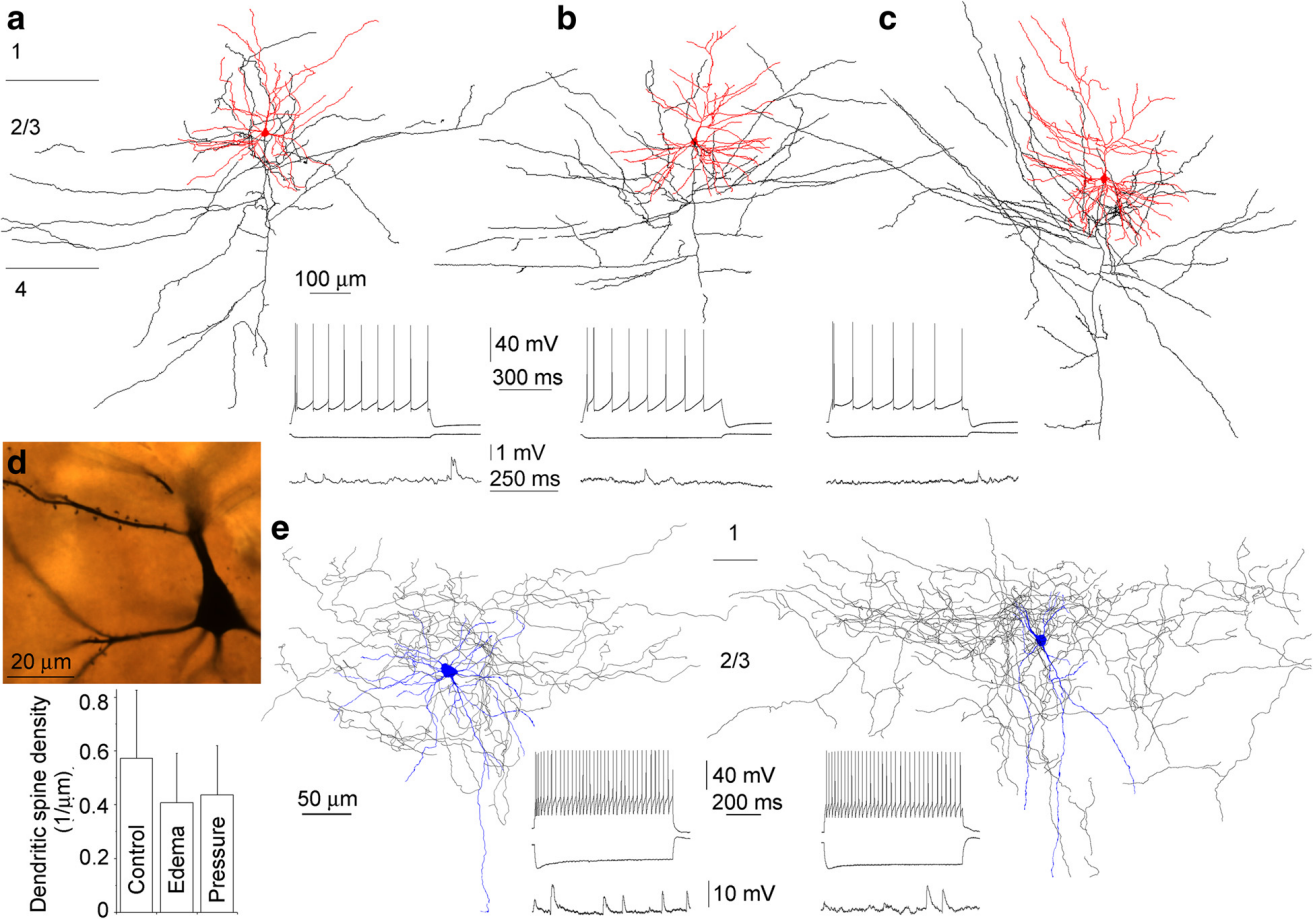
Electrophysiological and morphological characteristics of human neurons analysed by single cell digital PCR. Somatodendritic (*red*) and axonal (*black*) arborization of layer 2/3 pyramidal cells recorded in brain slices prepared from the Control (**a**), Edema (**b**) and Pressure (**c**) groups of patients. Insets display voltage responses of the cells to hyperpolarizing and depolarizing current pulses with characteristic regular spiking firing patterns (*top*) and representative periods of spontaneous subthreshold activity. Light microscopic image of the perisomatic region of a layer 2/3 human pyramidal cell recovered following electrophysiological recordings and cytoplasm harvests (**d**, *top*). Measurements of the density of dendritic spines uncovered a significant drop in the Edema and Pressure groups relative to Control (bottom). Morphology and firing of human fast spiking interneurons cells used for single cell digital PCR (**e**). Somatodendritic (*blue*) and axonal (*black*) arborization of layer 2/3 fast spiking cells recorded in brain slices prepared from the Control (*left*) and Edema (*right*) groups of patients. Insets show voltage responses of the cells to hyperpolarizing and depolarizing current pulses with characteristic fast spiking firing patterns (*top*) and representative periods of spontaneous subthreshold activity

The remainder of the tissue blocks used for microarray analysis and QRT-PCR validation was cut for acute brain slice preparations (with *n* = 2, 12 and 6 additional slice experiments in Control, Edema and Pressure groups, respectively) in order to record human pyramidal cells and fast spiking interneurons in layer 2/3 using the whole cell patch clamp mode. Differential interference contrast microscopy was used to select putative pyramidal cells and interneurons based on perisomatic morphology, and the identity of pyramidal cells and interneurons was first confirmed according to their regular spiking and fast spiking firing characteristics in response to depolarizing current pulses, respectively (Fig. 1). Analysis of basic electrophysiological properties revealed that pyramidal cells in the Edema (*n* = 148) and Pressure (*n* = 125) groups had more depolarized resting membrane potentials compared to the Control (*n* = 55) group (−80.4 ± 5.3, −81.2 ± 5.6 and −83.3 ± 6.6 mV, respectively, *p* < 0.03) and had higher input resistances and more depolarized action potential threshold potentials in the Edema group (72.9 ± 39.3 MΩ and −46.7 ± 6.3 mV) compared to the Control and Pressure groups (47.7 ± 18.8 and 53.2 ± 33.4 MΩ, −50.9 ± 5.8–48.9 ± 5.8 mV, respectively, *p* < 0.001). Fast spiking interneurons were recorded only in the Control (*n* = 27) and Edema (*n* = 47) groups and were more depolarized at rest in edema (−74.9 ± 4.8 vs. −68.9 ± 5.7 mV, *p* < 0.001). Interneurons had higher input resistances and similar action potential threshold potentials in the Edema group (140.7 ± 46.2 MΩ and −44.3 ± 7.1 mV) compared to the Control group (127.7 ± 17.2 MΩ, *p* < 0.001 and −46.2 ± 5.1 mV, respectively). In addition, we observed a decrease in the frequency of spontaneous EPSPs arriving to human pyramidal and fast spiking cells in the Edema (0.51 ± 0.39 and 5.96 ± 3.79 Hz, respectively) and to pyramidal cells in the Pressure (0.50 ± 0.28 Hz) groups relative to the Control group (1.51 ± 0.82 and 10.01 ± 3.19 Hz, *p* < 0.03 and *p* < 0.001, respectively) without a change in spontaneous EPSP amplitudes (pyramidal cells, 0.66 ± 0.40, 0.61 ± 0.30, 0.79 ± 0.37 mV; interneurons, 2.45 ± 0.67, 2.31 ± 0.71, respectively, Fig. 1.).

Post hoc anatomical recovery and analysis of dendritic and axonal arborizations further ensured the identity of pyramidal cells and classified interneurons as basket cells based on axonal terminals frequently observed adjacent to unlabeled somata (Fig. 1). To test potential morphological alterations in pathological conditions, we first compared the length of apical and basal dendrites and the length of axons remaining in the slices based on three-dimensional reconstructions human pyramidal cells recorded in the Control (*n* = 5; 3845 ± 2123, 3829 ± 1840, 17201 ± 9563 μm, respectively), Edema (*n* = 10; 3987 ± 1157, 4817 ± 1854, 16780 ± 7272 μm, respectively) and Pressure (*n* = 5; 4528 ± 1384, 4861 ± 1591, 1629 ± 3985 μm, respectively) groups, however, we detected no significant differences (*p* ≥ 0.12). Dendritic spines are dynamically altered in response to functional changes [4], but are remarkably stable in adulthood [2]. Earlier experiments found that the density of dendritic spines differs between frontal and parietal areas in primates [11] and developmentally stabilizes in adulthood around the average age of patients involved in our study in humans [32]. When measuring the density of spines on dendritic branches of basal and oblique dendrites at distances between 70 and 150 μm from the soma in samples taken from the prefrontal gyrus, we found that spine densities on dendrites of human pyramidal neurons in the Edema (*n* = 38, 0.38 ± 0.18/μm) and Pressure (*n* = 30, 0.43 ± 0.17/μm) groups were smaller of what we found on pyramidal cells of the Control (*n* = 31, 0.55 ± 0.23/μm, respectively, *p* < 0.028) group (Fig. 1). Spines receive the majority of glutamatergic input arriving to pyramidal neurons and the drop in spine densities indicate a loss of excitatory synapses in disease and, in turn, lower synapse numbers might contribute to higher input resistances measured in edema. The morphology of individual dendrites and length of the dendritic arborization of basket cells in the Control (*n* = 3, 4155 ± 804 μm) and Edema group (*n* = 3, 4497 ± 741 μm) appeared similar, axonal lenghts were not measured due to a high number of thick branches cut at the surface of slices.

**Fig. 2:**
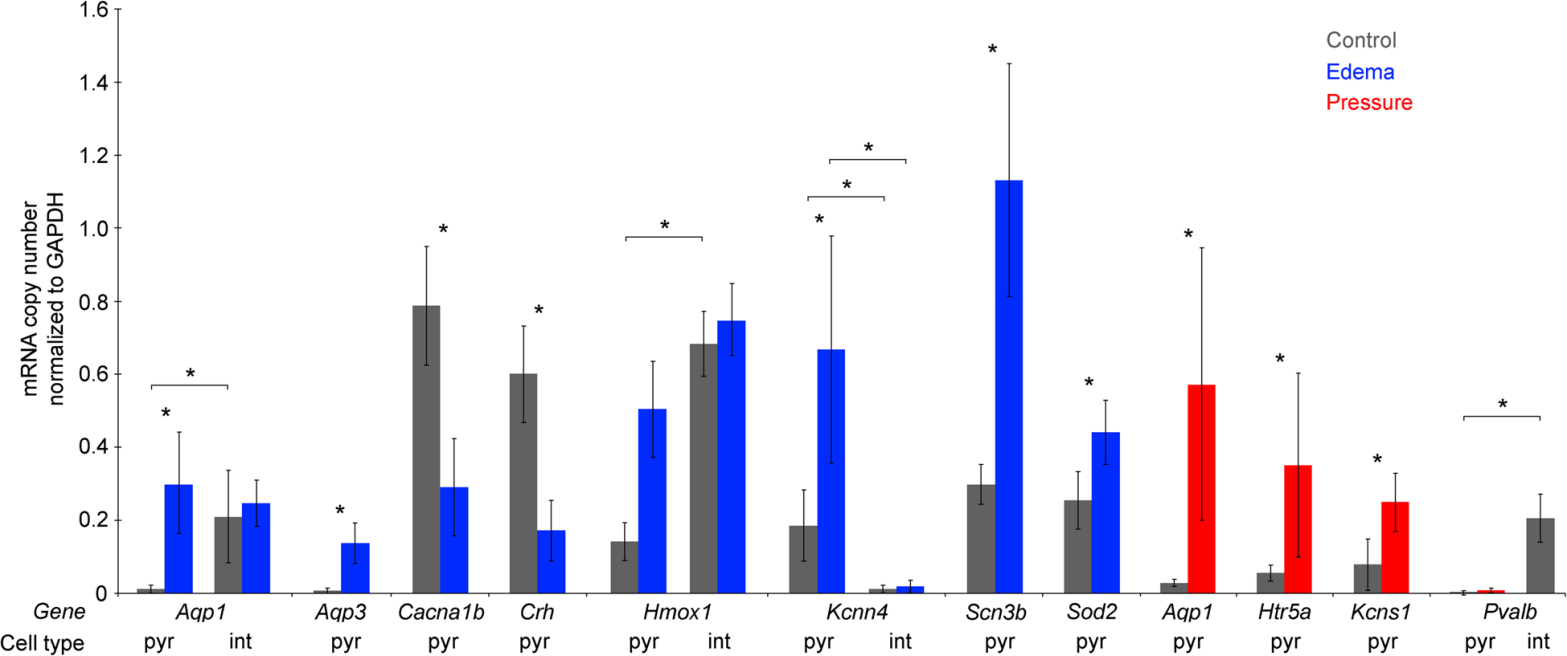
Results of single cell digital PCR performed on previously patch clamp recorded human pyramidal cells (pyr) and fast spiking interneurons (int). Groups of patients are colour coded, asterisks indicate significant (*p* < 0.05) differences

Following electrophysiological characterization, we collected cytoplasm samples from the recorded pyramidal cells to verify mRNA expression changes detected homogenized tissue samples. Aiming for a precision of single mRNA molecules in quantification, we performed single cell reverse transcription and digital PCR on cytoplasm samples aspirated into the recording pipette adapting our method developed for rat neurons [13, 23]. We selected 12 genes for testing mRNA copy numbers in single pyramidal neurons (*n* = 88) and fast spiking interneurons (*n* = 31), using three to six cells for each gene comparing Control versus Edema or Pressure groups of patients (Fig. 2). Copy numbers of the selected genes were normalized to those of the homeostatic glyceraldehyde-3-phoshate dehydrogenase *GAPDH* gene determined in the same cells. As expected, *GAPDH* was expressed in similar copy numbers in individual neurons across Control, Edema and Pressure goups in pyramidal cells (54 ± 13, 53 ± 15 and 57 ± 14, respectively) and in interneurons of Control (55 ± 8) and Edema (51 ± 9) groups. Human pyramidal cells showed significant mRNA copy number changes in agreement with data from homogenized tissue in 11 out of 12 genes. Single neuron digital PCR validated changes were detected in genes coding cytoprotective enzymes (*HMOX1*, *SOD2*) voltage-gated ion channels (*CACNA1B*, *KCNN4*, *KCNS1*, *SCN3B*), aquaporins (*AQP1*, *AQP3*), serotonin receptor 5A (*HTR5A*) and corticotrophin releasing hormone (*CRH*). Moreover, parvalbumin (*PVALB*), known to be expressed in GABAergic interneurons but not in pyramidal cells, could not be detected in copy numbers above noise level in human pyramidal cells in the Control and Pressure groups of patients, while fast spiking cells expressed *PVALB* in copy numbers of 12 ± 4 in the Control group (Fig. 2). The same primer set detected up-regulation of the *PVALB* gene in homogenized cortical samples in response to pressure suggesting cell type specific expression in nonpyramidal cells. In contrast to pyramidal cells, genes tested in fast spiking interneurons did not follow expression patterns predicted by results of homogenized tissue. Genes showing significant changes in tissue homogenates and pyramidal cells (*AQP1*, *HMOX1*) were unchanged in mRNA copy numbers in fast spiking cells collected from the Control or Edema groups of patients. In addition, *KCNN4* could not be detected above signal threshold in fast spiking cells in these patient groups. Thus, pyramidal cells and fast spiking interneurons showed similar copy numbers of homeostatic genes, however, some cytoprotective enzymes (*HMOX1*), aquaporins (*AQP1*) and voltage-gated ion channels (*KCNN4*) were differently expressed by pyramidal cells and fast spiking interneurons of control patients. Moreover, the expression of *HMOX1*, *AQP1* and *KCNN4* was altered in pyramidal cells but remained unchanged in fast spiking cells in response to edema suggesting cell type specific regulation in pathological conditions. Our single cell dataset limited to a subset of neuron types does not exclude the contribution of glia and endothel to tissue level changes or potential cell type specific alterations in non-neuronal cell types in Edema or Pressure groups of patients.

**Fig. 3:**
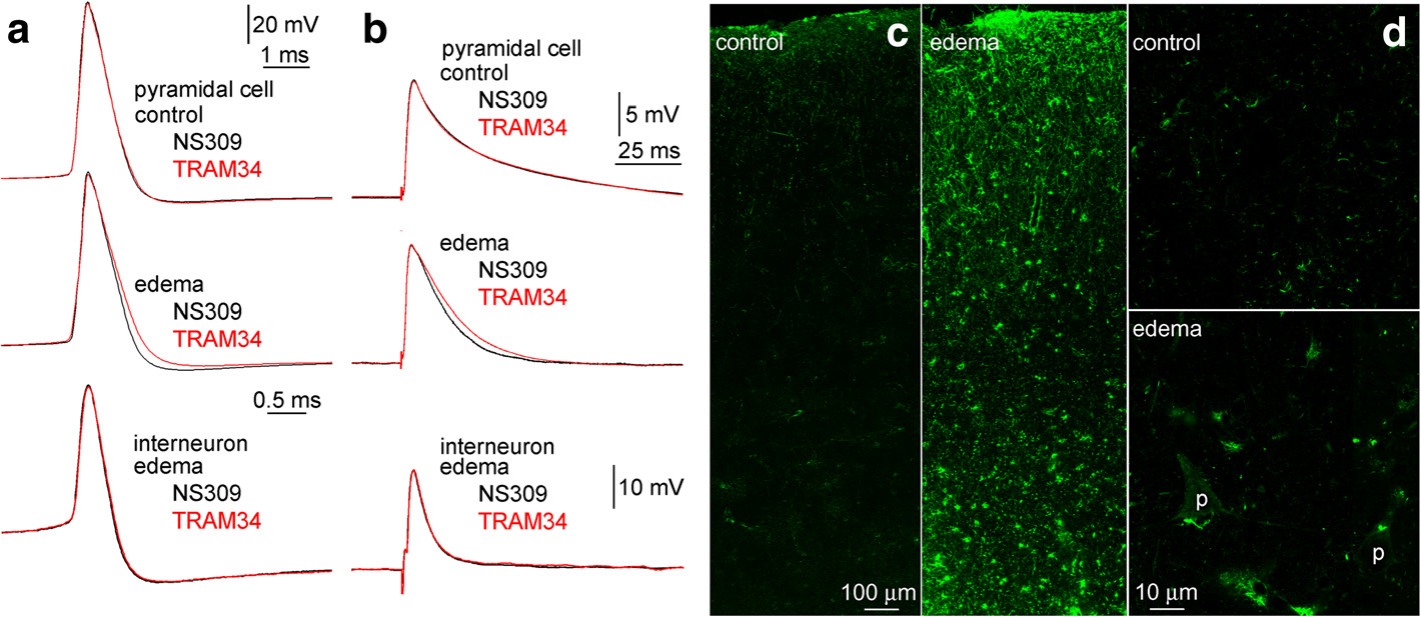
Functional validation of KCNN4 mRNA copy number changes in detected human pyramidal cells. Waveforms of action potentials evoked by depolarizing current injections (**a**) and of extracellularly evoked EPSPs (**b**) responded differently to the serial application of the small- and intermediate-conductance calcium activated potassium channel activator NS309 (500 nM) and TRAM34 (1 μM), an inhibitor of intermediate-conductance calcium activated potassium channels. The descending phase of action potentials and EPSPs was shortened in pyramidal cells recorded in brain slices prepared from the Edema group, but remained unchanged in pyramidal neurons of the Control group and in fast spiking interneurons of the Edema group. Traces shown are population averages. Confocal images of immunoreactions with antibodies against *Kcnn4* performed simultaneously on samples of the Control and Edema groups showing a cross section of the gray matter (**c**) and part of layer 3 similar to areas where electrophysiological experiments were performed (**d**). Pyramidal cells were not labeled in the Control group and moderate *Kcnn4* positivity was detected in pyramidal cells (p) of the Edema group. In addition, intense immunolabeling for *Kcnn4* was detected in glial cells resembling astrocytes and interlaminar glia in both groups of patients

To functionally validate some results showing changes in mRNA copy numbers in identified pyramidal cells, we have chosen *KCNN4* encoding the α-subunit of KCa3.1 channels. This follows earlier experiments in rat brain edema models suggesting neuroprotective effects of Kcnn4 blockers [20], presumably acting on Kcnn4 channels located on activated microglia [17] and immune-competent cells invading the brain [38]. The expression of Kcnn4 is well established on non-neuronal cells of the brain [17, 38], however, the presence of Kcnn4 channels in CNS neurons has only been recently demonstrated in rat Purkinje cells [12] and the expression or activity of Kcnn4 in neurons of the human CNS has not been documented. Thus, we tested the function of Kcnn4 on human pyramidal and fast spiking cells in slices cut from the Control and Edema groups first by introducing the small- and intermediate-conductance calcium activated potassium channel activator NS309 (500 nM) followed by the application of TRAM34 (1 μM), an inhibitor of intermediate-conductance calcium activated potassium channels (Fig. 3). The kinetics of action potentials and EPSPs of pyramidal cells (*n* = 10 from three patients) in the Control group was not changed by these pharmacological manipulations (0.616 ± 0.130 ms vs 0.608 ± 0.145 ms and 13.14 ± 2.05 ms vs 13.11 ± 2.10 ms, Wilcoxon test *p* = 0.284 and 0.937, respectively), however, the half-width of action potentials and EPSPs of human pyramidal cells (*n* = 12 from three patients) recorded in the Edema group was significantly altered by the same procedure (from 0.584 ± 0.174 ms to 0.681 ± 0.209 ms and from 13.43 ± 2.40 ms to 15.76 from 0.584 ± 0.174 ms to 0.681 ± 0.209 ms 2.42 ms, Wilcoxon test *p* < 0.005 and 0.006, respectively). Such alterations in action potential (0.334 ± 0.066 vs. 0.341 ± 0.071 ms) or EPSP (7.51 ± 2.47 vs. 7.54 ± 2.56 ms) half widths were not observed when testing fast spiking interneurons (*n* = 9) in the Edema group in agreement with single digital PCR results showing no expression of *KCNN4* in interneurons. Moreover, we tested the presence of Kcnn4 as a protein with immunocytochemistry using antibodies against Kcnn4 performed simultaneously on samples of the Control (*n* = 3) and Edema (*n* = 3) groups (Fig. 3). This resulted in no detectable signal in pyramidal cells of the Control group and moderate Kcnn4 positivity in pyramidal-like cells of the Edema group, however this was accompanied by strongly Kcnn4 immunopositive small, presumably glial cells and the identity of which could not be verified due to suboptimal technical possibilities associated with the human tissue and antigen retrieval methods.

## Discussion

The workflow presented above paves the way for detecting neuron type specific changes at molecular, electrophysiological and morphological level in various pathological conditions in the human brain. Cell type specific therapy targeted against key molecular components might form the basis in future treatment of psychiatric or neurological disorders [15, 19]. Emerging differences between the human cerebral cortex and its animal models widely used in experimentation [24, 43] might contribute to factors leading to failing clinical trials in the field [15] and argue for the use of human samples. In spite of recent treatment targets for cerebral edema and ICP including the Na^+^-K^+^-2Cl^−^ co-transporter (NKCC1) and the SUR1-regulated NC_Ca-ATP_ (SUR1/TRPM4) channel [44], osmotherapy remains typically administered [36, 44]. Some of the edema/ICP modulated and single human pyramidal cell verified gene products identified here might be considered as novel pharmacological targets in cell type specific neuroprotection. For example, aquaporins were suggested to be crucial in extracellular space- and osmoregulation in the brain [18, 26] and are reportedly expressed in astrocytes (*AQP4* and *AQP9*) [10, 29] and in the epithelium of the choroid plexus (*AQP1*) [30], but neuronal aquaporin expression is not clear. In agreement with earlier reports [10, 18, 29, 30], we detected no expression of aquaporins in pyramidal cells of the Control group, however, human pyramidal cells contained *AQP1* and *AQP3* mRNA in edema and *AQP1* in patients with increased ICP, suggesting a redistribution of water (*AQP1*) and water plus glycerin permeable (*AQP3*) aquaporins in the cerebral cortex in response to disease. Furthermore, human glia-derived brain tumors (gliomas, glioblastomas, meningiomas and schwannomas) do not express *AQP3* [31] arguing for a potentially neuron specific expression. It remains to be elucidated whether potentially interneuron selective expression of *AQP1* in the Control group contributes to neuron type selective water homeostasis. Differential sensitivity of different neuronal and glial populations in various types of head injury is well documented [7, 37] and cell type specific analysis under these conditions might identify underlying pathomechanisms leading to prevention or treatment.

Pharmacological and immunohistochemical results confirm our single cell digital PCR data and suggest a functionally detectable presence of *KCNN4* and KCa3.1 channels in edema relative to control conditions in human pyramidal cells, however, these channels are absent in fast spiking interneurons in both groups of patients. Moreover, our results support earlier findings functionally linking *KCNN4* and edema [20] and raise the possibility that the functional expression of Kcnn4 changes from purely glial to glial and neuronal in control and edemic conditions, respectively. A potential neuroprotective function of Kcnn4 channels on human pyramidal cells in edema might be the contribution to the suppression of EPSP summation and to the curtailment of action potentials leading to lower Ca^2+^ entry and excitotoxicity [12]. Speculatively, this effect of Kcnn4 might counteract Ca^2+^ influx due to large amplitude EPSPs resulting from the increased input resistance and rising from a more depolarized resting membrane potential in edema. The cell type dependent absence of this mechanism suggests differential survival of neuron populations in edema making fast spiking interneurons especially vulnerable when responding to extremely powerful unitary EPSPs driving these cells to fire in human microcircuits [24]. In addition, voltage-gated Ca^2+^ channels, like Ca_v_2.2 conducting N-type Ca^2+^ currents encoded by the *CACNA1B* gene found here to be down-regulated in human pyramidal cells in edema, are essential in action potential to release coupling at presynaptic terminals [6, 40] and could effectively suppress excitatory output and counteract excitotoxicity. Indeed, blockade of N-type VGCCs decrease neuronal damage associated with ischemic brain injury in animal models [42] and the *CACNA1B* gene is associated with cerebral infarction in a human population [45]. The voltage-gated Na^+^-channel β3 subunit encoding gene *SNC3B* found to be up-regulated in human pyramidal cells in edema might also be involved mechanisms governing excitability. Studies in *Scn3b*^−/−^ mice revealed spontaneous cardiac arrhythmia and conduction abnormalities [14] and the recently uncovered structure of trimeric β3 subunits of Na^+^-channels together with their suggested interactions with α subunits [27] predict future testing of β subunit dependent μ-conotoxin sensitivity of Na^+^-channels [47] in disease or treatment. Pyramidal neuron protective regulation of *KCNN4*, *CACNA1B*, and *SCN3B* suggested here could be effectively boosted by other cytoprotective processes involving heme oxygenase 1 [16] and mitochondrial superoxide dismutase 2 encoded by the *HMOX1* and *SOD2* genes which are up-regulated in pyramidal neurons in edema. The voltage-gated K^+^-channel α subunit Kv9.1 encoded by the *KCNS1* gene up-regulated in human pyramidal cells in the Pressure group has been found to be associated with multiple chronic pain states [9], however, interpretation of our findings is difficult knowing that electrically silent Kv9.1 subunits are not capable of forming functional homo-multimeric channels [39] but can suppress currents mediated by Kv2 and Kv3 α subunit families [3].

## Conclusions

We applied the combination of cellular electrophysiology, anatomy and single cell digital PCR to human neurons identified in brain slices in situ to assess mRNA expression and corresponding functional changes in response to edema and increased intracranial pressure. In material derived from patients with edema, mRNA copy numbers of AQP1, AQP3, HMOX1, KCNN4, SCN3B and SOD2 increased, while CACNA1B, CRH decreased in individual pyramidal cells, however, AQP1, HMOX1and KCNN4 remained unchanged in single cell digital PCR performed on fast spiking cells. In single pyramidal cells derived from patients with increased intracranial pressure, the copy number of AQP1, HTR5A and KCNS1 mRNAs was increased. Pharmacological and immunohistochemical results also suggested the presence of KCNN4 encoding the α-subunit of KCa3.1 channels in edema on pyramidal cells, but not on interneurons validating single cell digital PCR data. The frequency of spontaneous EPSPs decreased on pyramidal cells in both pathophysiological conditions and on fast spiking interneurons in edema. This was parallelled by an increase in input resistances on both cell types and by a drop in dendritic spine density on pyramidal cells consistent with a loss of excitatory synapses. The anatomical and/or physiological changes in individually identified human pyramidal and fast spiking cells in edema and increased intracranial pressure reveal cell type specific quantitative changes in gene expression and some of the modulated gene products identified here might be considered as novel pharmacological targets in cell type specific neuroprotection.
